# An Identification Method for Road Hypnosis Based on the Fusion of Human Life Parameters

**DOI:** 10.3390/s24237529

**Published:** 2024-11-25

**Authors:** Bin Wang, Jingheng Wang, Xiaoyuan Wang, Longfei Chen, Chenyang Jiao, Han Zhang, Yi Liu

**Affiliations:** 1 College of Electromechanical Engineering, Qingdao University of Science and Technology, Qingdao 266000, China; wangbin@mails.qust.edu.cn (B.W.); chenlongfei@mails.qust.edu.cn (L.C.); jiaochenyang@mails.qust.edu.cn (C.J.); zhanghan@mails.qust.edu.cn (H.Z.); yiliu@mails.qust.edu.cn (Y.L.); 2 Department of Mathematics, Ohio State University, Columbus, OH 43220, USA

**Keywords:** road hypnosis, human life parameters, state identification, vehicle, drivers

## Abstract

A driver in road hypnosis has two different types of characteristics. One is the external characteristics, which are distinct and can be directly observed. The other is internal characteristics, which are indistinctive and cannot be directly observed. The eye movement characteristic, as a distinct external characteristic, is one of the typical characteristics of road hypnosis identification. The electroencephalogram (EEG) characteristic, as an internal feature, is a golden parameter of drivers’ life identification. This paper proposes an identification method for road hypnosis based on the fusion of human life parameters. Eye movement data and EEG data are collected through vehicle driving experiments and virtual driving experiments. The collected data are preprocessed with principal component analysis (PCA) and independent component analysis (ICA), respectively. Eye movement data can be trained with a self-attention model (SAM), and the EEG data can be trained with the deep belief network (DBN). The road hypnosis identification model can be constructed by combining the two trained models with the stacking method. Repeated Random Subsampling Cross-Validation (RRSCV) is used to validate models. The results show that road hypnosis can be effectively recognized using the constructed model. This study is of great significance to reveal the essential characteristics and mechanisms of road hypnosis. The effectiveness and accuracy of road hypnosis identification can also be improved through this study.

## 1. Introduction

Traffic accidents are one of the major transportation issues faced by modern society. It is indicated that 70% of all traffic accidents are caused by driver-related factors [1]. Among these, 25% of traffic accidents are attributed to distracted driving, and 20% are caused by fatigued driving [2,3]. In 1963, Williams discovered that when drivers maintain a correct driving posture and travel on monotonous road environments, they tend to experience a state like hypnosis [4]. Williams et al. posited that this hypnosis-like state manifests as drivers fixating on the road lines ahead or at a fixed point, which renders them unable to assess hazardous situations during the driving process and respond appropriately in a timely manner [5]. Brown further explained that even when drivers maintain the correct posture, keep their eyes on the road ahead, and hands on the steering wheel, they may experience a hypnosis-like state [6]. A phenomenon known as “sleeping with the eyes open during driving” is proposed in a research report titled *Sleeping with the Eyes Open*. It is noted in the report that under specific circumstances, automobile drivers may fall into the peculiar state of “sleeping with the eyes open” [7]. According to the survey, accidents resulting in death caused by vehicle incidents account for about one-third of all traffic-related fatalities in the United States. Among these accidents, 50% are caused by personal factors such as fatigue and distraction [8]. Hanlon and Kelley recorded an objective state of drowsiness in drivers during an open-road driving experiment conducted in 1977. In their experiment, drowsy drivers were seated in a truck equipped with an electroencephalogram (EEG) recording device. A safety observer ensured the safety of the experiment by controlling the vehicle’s steering and braking. EEG data showed that, while driving on a straight section of the highway, the drivers entered a sleep-like state lasting up to 15 s. The vehicle often swerved between multiple lanes, yet no collision occurred. Ultimately, without the reminder from the safety observer, drivers might have deviated from the road during prolonged microsleep episodes [9]. Miller described these phenomena after analyzing 10,000 h of EEG data from truck drivers and stated that they represented periods when drivers’ attention shifted from the current task to an internal focus [10]. Kerr introduced the concept of “Driving Without Awareness (DWA)” and considered that the primary characteristic of this state is the loss of driver awareness caused by highly predictable visual scenes [11]. Through virtual driving experiments, Briest found that some drivers fall into a deep DWA state on monotonous environments like highways, characterized by a significant loss of awareness, during which inattentive driving patterns also emerge [12].

Xiaoyuan Wang conducted exploratory research on road hypnosis and described it as an unconscious driving state caused by a combination of external environmental factors and the drivers’ psychological state [13,14,15]. This state arises from the repetitive and low-frequency stimuli present in highly predictable driving environments. It manifests as sensory numbness, decreased attention, and reduced vigilance and may include transient states of confusion, amnesia, and hallucinations. This state can be induced by various factors, such as endogenous factors (the drivers’ susceptibility to hypnosis, fatigue, and circadian rhythms) and exogenous factors (road geometry, monotony of the driving task, monotony of the driving environment, and the enclosed nature of the vehicle). Drivers typically experience an obvious state of alertness when they emerge from road hypnosis. Although drivers often cannot remember what occurred during the state of road hypnosis, they can clearly recall the preceding dazed condition. While drivers in this state appear to maintain normal driving behavior, their reaction times are significantly slower than in normal driving conditions. Virtual driving experiments and vehicle driving experiments are designed and implemented to collect electrocardiogram (ECG) and electromyogram (EMG) signals, which are then integrated to develop a model for identifying the state of road hypnosis.

EEG is a physiological signal that records brain activity. The electrical activity of neurons is obtained by electrodes placed on the scalp [16]. Wertheim discovered that physiological characteristics such as eye movement information and changes in EEG signals can be used to determine whether a driver has reached a state of hypnosis [17]. Brown et al. used physiological information such as eye movements and heart rate from drivers to establish a model for fatigue driving identification [18]. Balasubramanian et al. analyzed EEG data to evaluate the cognitive fatigue state of drivers [19]. Awais found that the power levels in the Alpha and Theta frequency bands significantly increase when a person shifts from an alert state to a fatigued state. This change is more pronounced in the occipital and parietal regions compared to other areas [20]. Borghini found that drivers exhibit increased theta activity and reduced alpha activity in their brain activity when faced with high workload tasks [21]. Currently, there is no research on road hypnosis identification with EEG data. However, EEG data have been used to determine the state of hypnosis in medical and other research fields. Gorton found that the EEG recorded during hypnosis is like that recorded during the awake state and different from that recorded during sleep [22]. Nancy found that EEG can be utilized to evaluate an individual’s susceptibility to hypnosis. During the actual process of hypnotic induction, a significant increase in theta wave energy is observed in the posterior cortex, along with an increase in alpha activity across all regions [23]. Cerezuela measured EEG signals in highly predictable driving environments compared to less predictable ones. Their research indicated that drivers unconsciously experience a hypnotic state in the former condition, with reduced EEG levels [24]. Anoushiravan compared the effects of hypnosis alone and hypnosis with post-hypnotic suggestions on the Stroop effect and its facilitative and inhibitory components. The mechanisms of hypnosis at the neural level were investigated through the analysis of EEG frequencies. EEG recordings from the Stroop task revealed that participants under the influence of hypnosis exhibited significant increases in θ and β energy in their frontal lobes [25]. Golnaz B. Alejandro assessed the EEG brain activity of participants with high or low hypnotizability scores to understand the levels of hypnotizability reflected in these EEG activities [26].

Eye-tracking technology has become an effective method for detecting driver fatigue and distraction [27,28]. Sonle discovered that eye movements can serve as a measure of cognitive distraction by predicting and observing differences in eye movements [29]. Mackenzie found that drivers who perform well on cognitive tasks also exhibit more effective eye movement strategies during driving [30]. Horng established a driver fatigue detection system with eye-tracking technology, which achieves an identification efficiency of up to 90% [31]. Palinko found that driver cognitive load can be reliably estimated with eye movement information through experiments [32]. Aziman found through experiments that the drivers’ fixation time is significantly shortened, and the pupil diameter is significantly increased during distracted driving [33]. Xu designed a non-intrusive fatigue driving assessment system with eye-tracking technology and found significant differences in the threshold distribution of the pupil area between normal and fatigued driving states [34]. Andre discovered that spontaneous blink rate (BR) significantly and strongly decreases under fatigue conditions [35]. Miyaji collected driver eye parameters using stereoscopic cameras in a virtual driving environment and used them as feature parameters to identify cognitive distractions in driving behavior. They proposed an AdaBoost-based method for identifying cognitive distraction in drivers. The experimental results showed that the selection of eye parameters significantly improved the accuracy of cognitive distraction identification [36]. Antoine extracted blink features from eye movement signals and used fuzzy logic to fuse the extracted features to establish an EOG-based drowsiness detector [37].

Currently, EEG signals and eye movement data are mainly used in research to identify abnormal driving states, and no studies have specifically addressed methods for detecting road hypnosis. In this study, vehicle driving experiments and virtual driving experiments are designed to collect eye movement data and EEG signals from drivers. The Butterworth filter and Chebyshev filter are used to preprocess the eye movement data and EEG signals, respectively. Principal Component Analysis (PCA) and Independent Component Analysis (ICA) are applied to the preprocessed eye movement data and EEG signals for feature extraction. The SAM and DBN algorithms are used to construct the SAM model and DBN model separately, respectively. The SAM model and DBN model are integrated with SVM as the meta-model, and the stacking method is used to construct the road hypnosis state identification model for drivers. The experimental results showed that the SAM-DBN model, which integrates eye movement and EEG data, achieved higher accuracy and better generalization ability.

## 2. Experiment Methodology

### 2.1. Experiment Participants

**Figure 1:**
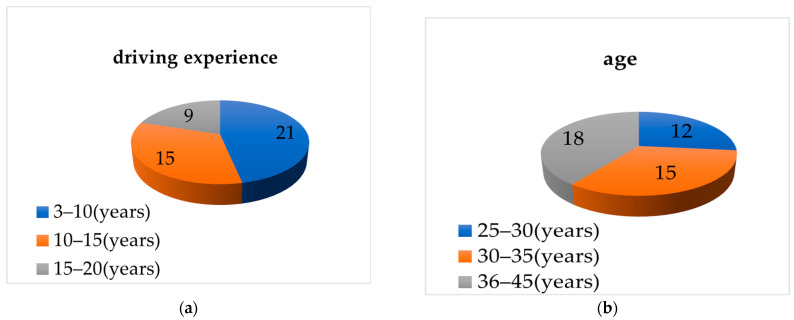
Basic information about the driver. (**a**) Driving experience. (**b**) Age.

Preliminary research on the state of road hypnosis revealed that experienced drivers are more prone to road hypnosis compared to novice drivers [13,14,15]. Participants in the experiment are required to have a vision of at least 600 degrees. A total of 45 drivers were recruited for the experiment, with a gender ratio of 8:2. Specific information is shown in Figure 1.

### 2.2. Experiment Equipment

**Figure 2:**
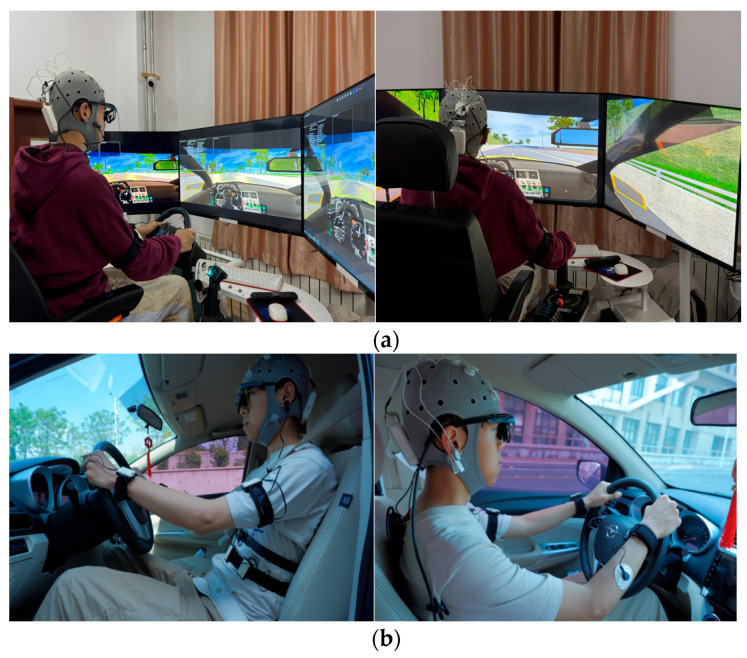
Experimental environment. (**a**) Virtual driving. (**b**) Vehicle driving.

Vehicle driving experiments and virtual driving experiments are included in this study. The virtual driving experiment platform consisted of a six-degree-of-freedom platform, a Logitech G29 steering wheel and pedals, three 55-inch high-definition displays, and Unity 3D software (version 2021.3.29f1c1). The vehicle driving experiments platform primarily consisted of a comprehensive road test vehicle, a laptop, and a video recorder. The environments for both vehicle driving experiments and virtual driving experiments are shown in Figure 2.

### 2.3. Data Collection Equipment

**Figure 3:**
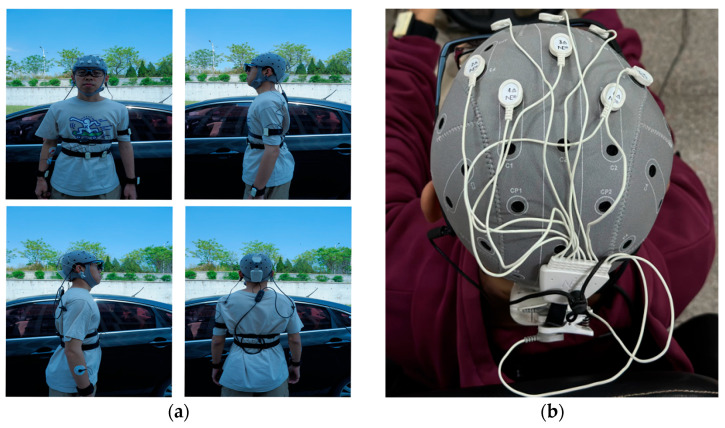
Experimental equipment. (**a**) Overall device wearing schematic diagram. (**b**) Schematic diagram of electrical channels in the brain.

Eye movement data are collected with the aSee Glasses from 7invensun (Beijing, China) Technology Co., Ltd. There are functions for the full experimental process that include eye movement recording, data analysis and visualization, and data export provided by the device. The EEG signals are collected with the Enobio Dx developed by Neuroelectrics Technology (Shanghai, China) Co., Ltd. The signal-to-noise ratio of the raw EEG signals can be restored by the device, and a perfect combination of high dynamic range is achieved. All DC signals are accurately recorded, and artifacts are eliminated. The experimental equipment is shown in Figure 3.

### 2.4. Procedure

#### 2.4.1. Vehicle Driving Experiments

Compared to virtual driving experiments, vehicle driving experiments involve many unstable factors in the driving route, such as sudden lane changes and overtaking. The experimental road is selected as the Qingdao Huangdao District Undersea Tunnel and Jiaozhou Bay Bridge to induce the drivers’ road hypnosis state as much as possible during vehicle driving experiments. The Jiaozhou Underwater Tunnel has a total length of 7.797 km, with the over-sea section being 4.095 km long. The road is designed as a six-lane urban arterial road with separated left and right lines and an elliptical cross-section. This is a monotonous, closed, straight-line tunnel section with a fixed interval of street lights as a fixed flash that can easily induce the driver to produce road hypnosis. The Jiaozhou Bay Bridge is 42.23 km long, with a speed limit of 80 km/h. The bridge is a six-lane, two-way road with a length of 31.63 km. The driving environment on the Jiaozhou Bay Bridge is relatively monotonous, with a “white noise” effect from the sea view, which can easily induce the driver to produce road hypnosis.

**Figure 4:**
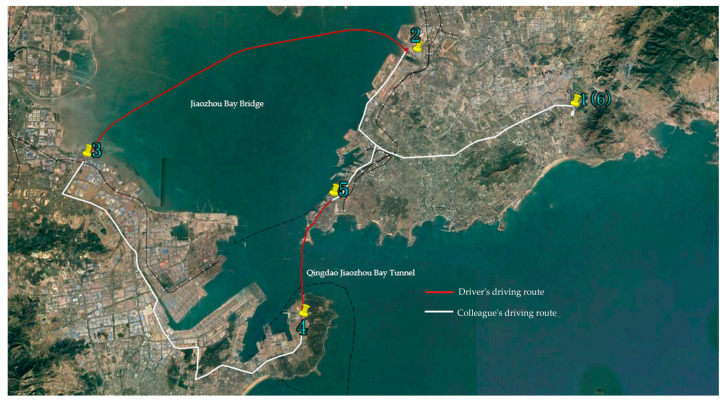
Vehicle driving experiment route.

Vehicle driving experiments are conducted from 9 a.m. to 12 p.m. Three assistants and three participants participated in the experiments. The driving speed is required to be maintained at 80 km/h as much as possible, and a constant speed is to be kept. Straight-line driving is to be maintained as much as possible to avoid lane changes and overtaking that could affect the experiment results. The specific experimental procedure is as follows:

(1) Before the experiment, an assistant equipped the driver with eye-tracking and EEG devices, connected these devices to a laptop, and secured the laptop in place. At the start of the experiment, this assistant recorded the start time and the total duration of the experiment;
(2) The route for vehicle driving experiments is shown in Figure 4. An assistant drove the vehicle from Point 1 to a stop near Point 2, where the experiment participant took over driving from Point 2 to Point 3, which included the Jiaozhou Bay Bridge. During the experiment, this assistant observed traffic and road conditions from the front passenger seat to ensure driving safety. Another assistant observed the drivers’ eye movement focus areas and changes in physiological signal data. When the driver’s eye focus area is fixed on a single point, or there are abnormal changes in the ECG signal, the assistant inquired if the driver is experiencing a state similar to hypnosis and recorded the time of the inquiry. After reaching Point 3, the participant rested for 15 min while the equipment was removed, checked, and adjusted, including battery levels. Then, an assistant drove the vehicle to a stop near Point 4, where the participant resumed driving and repeated the experiment procedure;
(3) After all participants’ data are collected, the data are exported from the software to a computer. An assistant drove the vehicle from Point 5 to Point 6, organized the experimental equipment, and concluded the experiment.

#### 2.4.2. Virtual Driving Experiments

Road hypnosis can be more effectively induced in virtual driving experiments. The experimental routes included a 50 km long, 15 km wide, four-lane straight road and a 20 km tunnel with fixed flashing points. The driving process excluded interference from other vehicles. Participants are required to have sufficient sleep before the experiments. The experiments started at 9:00 a.m. and ended at 12:30 p.m. There were three assistants who participated in the experiments in addition to the 45 participants. The experimental procedure is as follows:

(1) Before the experiment, an assistant adjusted the equipment and helped the driver wear the necessary devices. At the start of the experiment, this assistant recorded the start time of the experiment;
(2) During the experiment, the driver was required to maintain a speed of 120 km/h without changing lanes. The vehicle was turned around at the endpoint and the experiment lasted 30 min. One assistant continuously observed changes in the ECG signal, while another observed the eye movement equipment. If abnormal changes in the ECG signal or prolonged fixation in the driver’s eye movement were detected, the assistant would ask if the driver had experienced a state similar to hypnosis. The time of the inquiry is then recorded;
(3) After each experiment, an assistant asked the driver if a state similar to hypnosis had occurred during the driving process. For those who experienced a state similar to hypnosis, the event is recorded, and the driver is shown a video of the experiment to help recall if hypnosis had occurred with eye movement and physiological data for further verification. During this time, another assistant checked and adjusted the equipment. For drivers who experienced a state similar to hypnosis during the experiment, the experiment duration was extended to 40 min, and the procedure was repeated.

The experimental procedure was repeated until all experiments were completed. After the experiment, the collected data were exported from the software to a computer, the equipment was organized, and the experiment was concluded.

## 3. Data Processing and Discussion

After data collection, the data are organized according to the types of vehicle driving experiments and virtual driving experiments. This resulted in 45 sets of vehicle driving data and 45 sets of virtual driving data. Combining the experimental video and the characteristics of the data, 15 min of data with typical road hypnosis features are selected for each set by experts from the research team who have extensive experience in road hypnosis and driving behavior studies. The validity of the selected data is confirmed through expert evaluation. Eight drivers’ data are excluded due to attention distractions during the vehicle experiments. These distractions are caused by yawning, uncomfortable posture, and complex road traffic conditions. This resulted in 37 valid sets of vehicle driving data. In the virtual experiments, six sets of data are excluded. This left 39 valid sets of virtual driving data. The eye movement data are statistically analyzed. This analysis yielded 258,913 entries for vehicle experiments and 318,761 entries for virtual driving experiments. For EEG signals, 28,684 event-related potentials are marked. Among these, 12,513 are from vehicle driving, and 16,171 are from virtual driving. After the final experimental data are obtained, the eye movement data and EEG signals are preprocessed separately. The eye movement data are processed for outliers and filtered, while the EEG signals are subjected to selection, electrode localization, re-referencing, and filtering.

### 3.1. Data Preprocessing and Feature Extraction

#### 3.1.1. Eye Movement Data Preprocessing

(1) Data preliminary screening

The raw data collected by the eye tracker are exported. The eye movement data are marked as 1 for valid pupil recognition in both the left and right eyes and −1 for invalid data. Invalid data marked as −1 are removed. Additionally, data with a fixation point speed of less than or equal to 0 are also removed. The processed data are then checked for missing values. Rows with empty values are deleted to ensure the integrity and reliability of the data. During the data selection process, 15 min segments exhibiting hypnotic characteristics are selected based on the times when the drivers are asked questions and their responses. The remaining time periods are considered normal driving data. Outliers in the selected data are then processed. Outliers typically included data points that were significantly different from other observations or did not follow the expected pattern. In this study, the threshold for outliers is determined with the mean plus three times the standard deviation. This method considered the overall characteristics of the eye movement data and allowed for more accurate identification of outliers. Each column of the eye movement data is checked using this method. The process of processing outliers is shown in Algorithm 1.
**Algorithm 1.** Outlier DetectionInput: Datasets numOutput: Number of outliers in each column1: num columns = size (num, 2)2: num outliers = zeros (num columns, 1)3: for col = 1:num columns do4:    data=num (:, cool)5:    mean_data = mean(data) 6:    std_data = std(data) 7:    threshold = mean_data + 3 × std_data 8:    outliers = data > threshold 9:    num_outliers(col) = sum(outliers) 10:  fprintf(“Number of outliers in column ” + col + “: ” + num_outliers(col)) 11: end for

(2) Filtering

The filtered operation is applied to the preliminarily selected eye movement data to remove noise or unwanted components from the signal. Eye movement data usually contain a series of low-frequency components, such as the fixation duration at a point, which are crucial for subsequent analysis of eye movement behavior and the identification of driver hypnosis state. The Butterworth filter is chosen for this purpose because it aims for maximum flatness in the amplitude-frequency characteristics within the passband while providing rapid attenuation in the stopband. The Butterworth filter is a commonly used filter and belongs to the category of IIR (infinite impulse response) filters. It has a smooth frequency response and linear phase characteristics. The transfer function (frequency domain representation) of the Butterworth filter is given by the following formula:

(1) $$H(s)=\frac{1}{1+{\left(\frac{s}{\omega_{c}}\right)}^{2n}}$$

In this case, H(s) is the transfer function of the filter, s=jw is the complex variable frequency, j is the imaginary number unit, ω is the frequency, $\omega_{c}$ is the cut-off frequency, n is the filter order.

In this study, the Butterworth filter is designed as a fourth-order filter with a cut-off frequency of 5 Hz. High-frequency noise and other invalid information are present in the eye movement data. The fourth-order Butterworth filter is chosen for its excellent frequency response characteristics, which provide good frequency selectivity while avoiding excessive attenuation. The design process for the Butterworth filter is shown Algorithm 2.
**Algorithm 2**. Butterworth Filter DesignInput: Order, Cutoff frequency, Sampling frequency Output: Filter coefficients 1: order = 4 2: cutoff_frequency = 5 3: sampling_frequency = 120 4: normalized_cutoff_frequency = cutoff_frequency / (sampling_frequency / 2) 5: [b, a] = butter(order, normalized_cutoff_frequency, ‘low’)

After preprocessing, 158,947 entries of vehicle experiment data and 276,143 entries of virtual driving experiment data are obtained. In the vehicle experiment, each dataset included 15 min segments with road hypnosis characteristics. After preprocessing, 98,463 entries of road hypnosis data and 60,484 entries of normal driving data are obtained. In the virtual driving experiment, each dataset included 15 min segments with hypnosis characteristics. After preprocessing, 194,761 entries of road hypnosis data and 81,382 entries of normal driving data are obtained.

The preprocessed eye movement data are complex due to the inclusion of various types of information related to road hypnosis. Therefore, the data could not be directly used to construct a road hypnosis identification model. Key features need to be revealed with appropriate feature extraction techniques to facilitate the identification of physiological changes in drivers during the road hypnosis state.

Principal Component Analysis (PCA) is chosen for feature extraction from the eye movement data. PCA is a commonly used dimensionality reduction technique that can identify the main components or features in the eye movement data and project the data into a new feature space. The dimensionality of the data can also be reduced by PCA, redundant information can be eliminated, and the interpretability and processing efficiency of the eye movement data can be improved. Eye movement data are typically high-dimensional, and PCA can identify the directions of maximum variance in the data to extract the most representative features. The dimensionality reduction process of PCA effectively reduces the data’s dimensionality while retaining the most informative features.

The specific calculation process is as follows:

- The covariance matrix of the eye movement data is calculated. The covariance matrix describes the linear relationship between the data. The formula for calculating the covariance matrix is as follows:

(2) $$Cov(X,Y)=\frac{1}{n}\sum_{i=1}^{n}\left(X_{i}-\bar{X}\right)\left(Y_{i}-\bar{Y}\right)$$

In this case, X and Y represent two variables in the eye movement data respectively, different covariance matrices can be calculated by adding variables. $\bar{X}$ and $\bar{Y}$ represent the mean value of the variables X and Y, respectively.n is the number of samples;

b. The eigenvalues and corresponding eigenvectors are obtained by performing eigenvalue decomposition on the covariance matrix. The eigenvalues represent the variance in the eye movement data, while the eigenvectors represent the principal directions in the data. The formulas for calculating the eigenvalues and eigenvectors are as follows:

(3) Cov(X)v=λv

In this case, λ is the eigenvalue, and v is the corresponding eigenvector;

c. The largest K eigenvalues and their corresponding eigenvectors are selected as the principal components based on the magnitude of the eigenvalues. There is a K value that represents the number of principal components to be retained. This K value corresponds to the number of feature data points in the eye movement data.

#### 3.1.2. EEG Data Preprocessing

**Figure 5:**
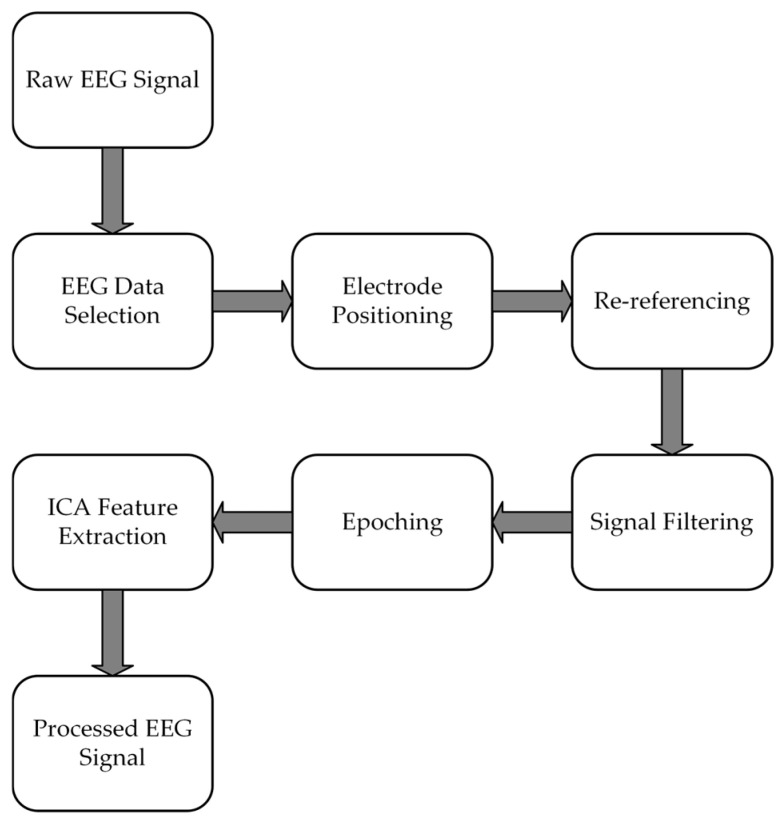
EEG pretreatment process.

The overall processing flow of EEG signals is shown in Figure 5:

In this study, EEG signals are collected with an eight-channel system. The channel names are Fp2, Fpz, Fp1, F4, Fz, F3, FC2, and FC1. The low-frequency pass is set to 0.1 Hz, and the high-frequency pass is to 250 Hz. Since EEG signals are difficult to label directly, abnormal fixation points from eye movement videos and the times when drivers are actively questioned during the experiment are used as the basis for classification. Event-related potentials (ERPs) during road hypnosis are labeled as “road hypnosis”, and ERPs during normal driving are labeled as “normal driving”. A total of 28,684 ERPs are marked, with 12,513 from vehicle driving and 16,171 from virtual driving. In vehicle driving, 7581 ERPs are labeled as road hypnosis, and 4932 are labeled as normal driving. In virtual driving, 9763 ERPs are labeled as road hypnosis, and 6408 are labeled as normal driving.

The specific processing steps are as follows:

(1) Electrode Localization:

Electrode localization involves mapping channel data, which refers to corresponding each EEG electrode channel to a specific position on the scalp (e.g., specific locations in the International 10–20 system). This localization determines the precise position of each electrode on the scalp. The specific steps are as follows:

- Attach EEG electrodes to the scalp according to the marking system guided by the International 10–20 system;
- Measure the potential distribution on the scalp with electrodes and record the signal corresponding to each electrode position;
- Use spatial interpolation to correspond these positions with the electrode channels in the EEG data.

(2) Re-referencing:

The average reference method is chosen for this study. This method compares each electrode’s signal with the average of all other electrodes’ signals and calculates the difference between each electrode’s signal and the average. This approach eliminates common mode interference between electrodes, reduces noise, and improves the signal-to-noise ratio. It makes the event-related potentials of “road hypnosis” and “normal driving” easier to observe and analyze.

(3) Filtering:

The Chebyshev filter is used to filter the EEG signals in this study. The Chebyshev filter is an IIR (Infinite Impulse Response) filter that provides limited ripple in both the passband and stopband and sharp cutoff characteristics. The Chebyshev filter is designed with a cutoff frequency of 30 Hz, a passband ripple limit of 1 dB, a stopband attenuation of 40 dB, and a filter order of 4. The transfer function of the Chebyshev filter is as follows:

(4) $$H(s)=\frac{1}{\sqrt{1+\varepsilon^{2}T_{n}^{2}\left(\frac{s}{\omega_{c}}\right)}}$$

In this case, H(s) is the transfer function of the filter. s is the complex variable in the frequency domain. s=jω, where j is the imaginary unit. ω is the frequency. $\omega_{c}$ is the cutoff frequency, which is the −3dB cut-off point of the filter. $T_{n}^{2}\left(\frac{s}{\omega_{c}}\right)$ is the Chebyshev polynomial and n is the order of the filter. ε is the ripple parameter, which is used to control the amount of ripples in the passband.

**Figure 6:**
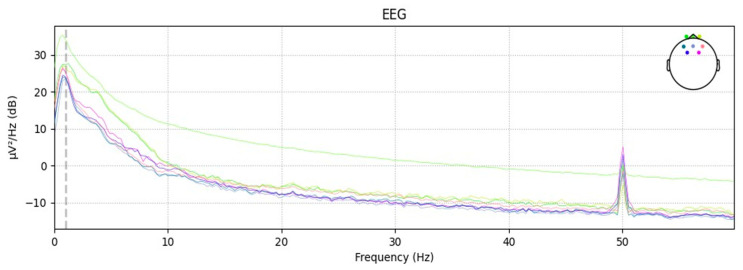
Filtered EEG power spectral density map.

In the transfer function formula of the Chebyshev filter, the ripple parameter ε can be used to control the amount of ripple in the passband. For processing EEG signals, a smaller ripple parameter is chosen to minimize ripple in the passband, allowing more accurate extraction and analysis of frequency components related to road hypnosis. Additionally, setting the filter order to fourth provides sufficient smoothness and stability, enabling more precise selective passing or suppression of signals within the target frequency range. Below is the power spectral density (PSD) plot of the EEG after filtering. The PSD plot reflects the power or energy distribution of the EEG signal across different frequencies, which is used to observe abnormal states in the EEG signals. Schematic diagram of EEG data after power spectral density processing is shown in Figure 6.

**Figure 7:**
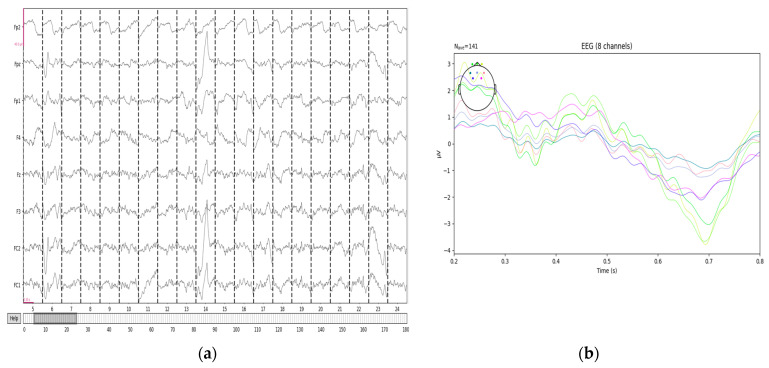
EEG segmented data. (**a**) EEG segmentation results for all channels. (**b**) The segmented results of 0.2–0.8 s.

Epoching refers to dividing continuous EEG signals into a series of fixed-length time windows (called epochs) to independently analyze and process the signals within each time window. Before epoching, the length of each time window needs to be determined. A length of t=0.2 s−0.8 s, or 0.6 s, is chosen. The continuous EEG signals are divided according to the set window length, with each epoch corresponding to the positions of the “road hypnosis” and “normal driving” labels. The 23rd set of EEG data from the vehicle driving experiment is divided into 180 epochs. The segmentation results are shown in Figure 7.

After preprocessing the EEG signals, 8972 segments with road hypnosis characteristics and 3541 segments of normal driving are identified in vehicle driving experiments. In the virtual driving experiment, 10,427 segments with road hypnosis characteristics and 5744 segments of normal driving are selected.

EEG signals are typically complex signals generated by multiple neural activities and cannot be directly used to construct a road hypnosis identification model. Suitable feature extraction techniques are required to independently separate the mixed signals. Independent Component Analysis (ICA) is commonly chosen for feature extraction from EEG signals. ICA assumes that the components of the signal are independent of each other. ICA effectively separates these independent components and identifies mutually independent EEG components. The specific calculation process is as follows:

The preprocessed EEG signals X are centralized by subtracting the mean of each feature, represented as follows:

(5) $$X_{c}=X-\mu$$

In this case, μ represents the mean value of the EEG signal.

By solving $W=A^{-1}$, the independent component matrix W is obtained, represented as follows:

(6) $$S=WX_{c}$$

In this case, $A^{-1}$ is the matrix that transforms the independent components back to the original data, S represents the extracted independent components of the EEG signals, W is the mixing matrix in ICA, $X_{c}$ is the centralized data.

**Figure 8:**
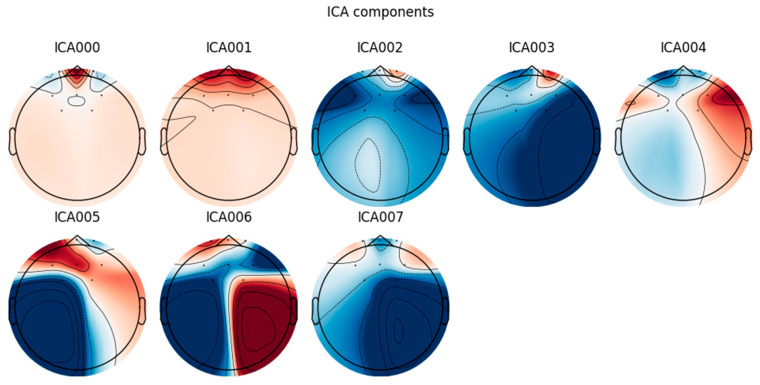
Schematic diagram of EEG data after ICA processing.

**Figure 9:**
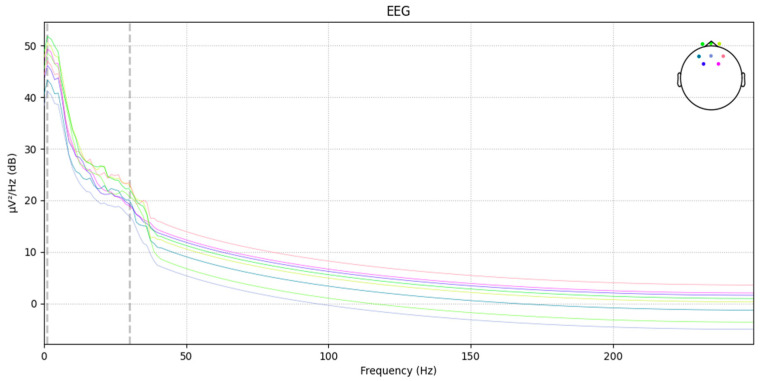
EEG power spectral density after ICA treatment.

The ICA method is used to effectively remove non-EEG independent components, such as eye movement artifacts and muscle movement artifacts. The results are shown in the Figure 8:

After performing Independent Component Analysis (ICA) on the EEG signals, the Power Spectral Density (PSD) plot of the EEG signals is shown in Figure 9:

### 3.2. Model

#### 3.2.1. Sam Model

**Figure 10:**
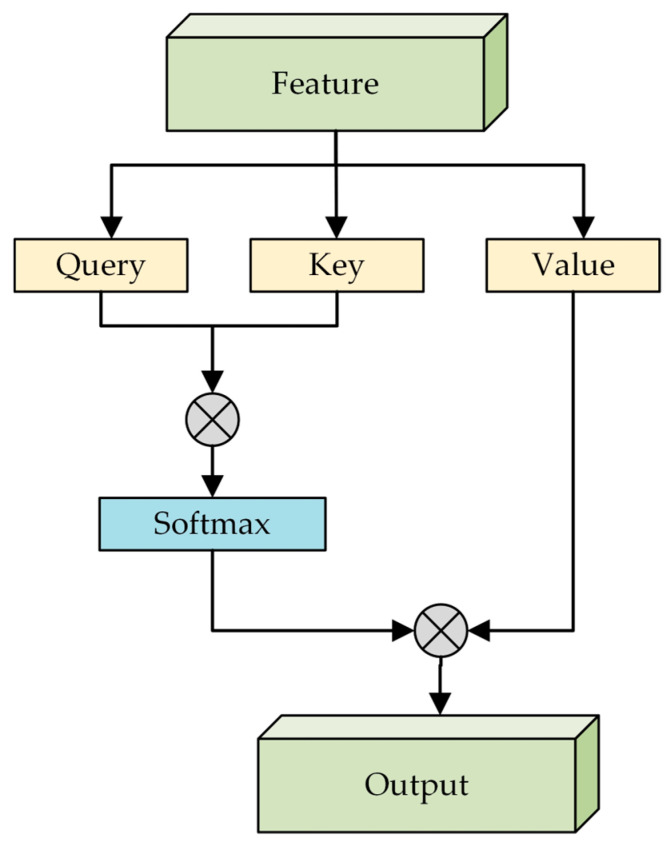
Self-Attention Models.

The Self-Attention Models (SAM) algorithm is chosen for this study. SAM includes four modules: self-attention mechanism, multi-head attention, residual connections, and layer normalization. The self-attention mechanism decomposes the input data into sub-parts and calculates the attention weights between them. Additionally, SAM has adaptability and flexibility, allowing it to automatically adjust weights based on different parts of the input data. The structure of SAM is shown in Figure 10.

The main calculation process of SAM is as follows:

(1) Attention Weight Calculation:

For each position i, the similarity with other positions j is calculated to obtain the attention weight $a_{ij}$

(7) $$a_{ij}=soft\max(\frac{q_{i}\cdot k_{j}}{\sqrt{d_{k}}})$$

In this case, $q_{i}=W_{q}x_{i}$, $k_{j}=W_{k}x_{j}$, $d_{k}$ is the dimension of *k*, $W_{q}$ and $W_{k}$ are the weight matrices.

(2) Weighted sum

The attention weights $\alpha_{ij}$ are used to perform a weighted summation of the vector representations for all positions, resulting in the context representation $c_{i}$ for each position.

(8) $$c_{i}=\sum_{j=1}^{n}\alpha_{ij}v_{j}$$

In this case, $v_{j}=w_{v}x_{j}$, $w_{v}$ is the weight matrix.

Calculate the output vector sequence:

(9) $$h_{n}=\sum_{j=1}^{N}v_{j}*a_{ij}$$

In this case, $ij\in \left[1,N\right]$ represents the positions of the output and input vector sequences and $a_{ij}$ denotes the attention weight from the i th input to the j th output.

#### 3.2.2. DBN Model

**Figure 11:**
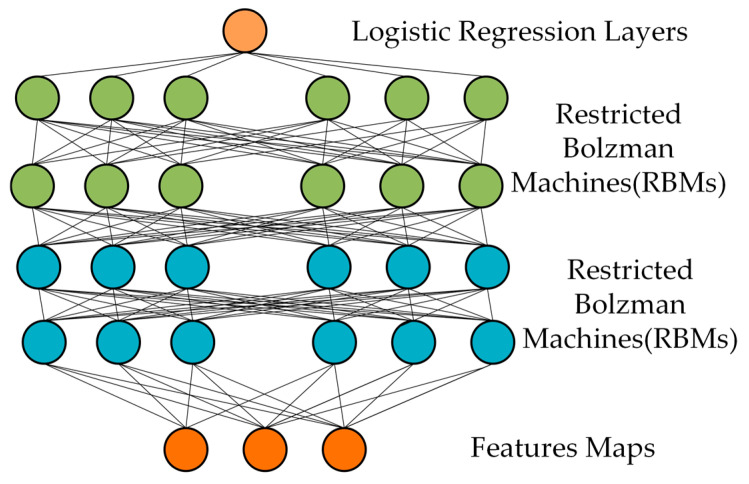
DBN model structure.

This study selects Deep Belief Networks (DBN) primarily due to their advantages in feature learning and hierarchical representation. DBN consists of five modules: restricted Boltzmann machines, visible layers, hidden layers, weight connections, and layer-wise training. DBN can automatically discover and represent hierarchical features through layer-wise learning and combination. DBN demonstrates good generalization capability when dealing with limited sample data. The structure of DBN is shown in Figure 11.

The computation process of DBN is as follows:

(1) Energy Function

The energy levels of different states are calculated by altering the states of the visible and hidden layers.

(10) $$E\left(v,h\right)=-\sum_{i=1}^{n}\sum_{j=1}^{m}W_{ij}v_{i}h_{j}-\sum_{i=1}^{n}a_{i}v_{i}-\sum_{j=1}^{m}b_{j}h_{j}$$

In this case, v is the state vector of the visible layer, and h is the state vector of the hidden layer. These states can be 0 or 1, which represent the activation status of the nodes. $W_{ij}$ represents the weights connecting the visible layer node i and the hidden layer node j, and $b_{j}$ denotes the bias terms for the visible and hidden layers.

(2) Joint Probability Distribution

The energy function is converted into a probability, which is then used for probability calculations during training and inference. The specific form is as follows:

(11) $$P(v,h)=\frac{1}{Z}e^{-E(v,h)}$$

In this case, z is the normalization factor, which ensures that the total sum or integral of the probability distribution equals 1.

(3) Marginal Probability Distribution

The marginal probability distribution is obtained by integrating or summing the joint probability distribution. This is used to compute the states of the visible or hidden layers. The specific form is as follows:

(12) $$P(v)=\sum_{h}P(v,h)$$

(13) $$P(h)=\sum_{v}P(v,h)$$

In this case, P(v) is the probability distribution of the visible layer states, and P(h) is the probability distribution of the hidden layer states.

#### 3.2.3. SAM-DBN Model

Model fusion methods primarily include simple averaging, weighted averaging, voting, Bagging (Bootstrap Aggregating), Boosting, and Stacking (Stacked Generalization). Table 1 describes the characteristics and applicability of each model fusion method.

This study used the Stacking method to combine the SAM model and the DBN model to create the SAM-DBN model. This method inputs the prediction results of the SAM and DBN models as features into a meta-model. The meta-model is responsible for integrating the predictions from both models and outputting the final fusion result. SVM is chosen as the meta-model. SVM is a non-parametric method that does not make specific assumptions about data distribution and can adapt flexibly to different types of data and models. For the SAM and DBN models, as well as the EEG and eye-tracking data used in this study, the non-parametric nature of SVM allowed for effective processing. Additionally, SVM classified the data by maximizing the margin, which provided good generalization capability. By using the predictions of SAM and DBN as input features, a more accurate overall prediction model is generated, thereby enhancing the model’s generalization ability. The predictions of SAM and DBN are used as input features to generate a more accurate overall prediction model, which improves the model’s generalization ability. The specific computation process is as follows:

(1) Solving the Convex Optimization Problem

Given the training dataset ${\left\{\left(x_{i},y_{i}\right)\right\}}_{i=1}^{N}$,

(14) $$\min_{w,b}\frac{1}{2}{\left\|W\right\|}^{2}+C\sum_{i=1}^{N}\xi_{i}$$

In this case, $x_{i}$ represents the feature vector and $y_{i}$ denotes the corresponding class labels.

Constraints:

(15) $$y_{i}\left(w\cdot x_{i}+b\right)\geq 1-\xi_{i},i=1,2,\ldots ,N$$

(16) $$\xi_{i}\geq 0,i=1,2,\ldots ,N$$

In this case, w is the normal vector of the hyperplane, b is the intercept of the hyperplane, $\xi_{i}$ is the slack variable, and C is the penalty coefficient.

(2) Transformation to Solve the Dual Problem

(17) $$\max_{\alpha }\sum_{i=1}^{N}\alpha_{i}-\frac{1}{2}\sum_{i=1}^{N}\sum_{j=1}^{N}y_{i}y_{j}\alpha_{i}\alpha_{j}x_{i}\cdot x_{j}$$

Constraints:

(18) $$0\leq \alpha_{i}\leq C,i=1,2,\ldots ,N$$

(19) $$\sum_{i=1}^{N}\alpha_{i}y_{i}=0$$

In this case, $\alpha_{i}$ is a set of optimal solutions.

(3) Classification Decision Function

(20) $$f\left(x\right)=sign\left(w\cdot x+b\right)$$

In this case, sign is the sign function which represents the class discrimination result.

### 3.3. Classification and Discussion of Road Hypnosis

**Figure 12:**
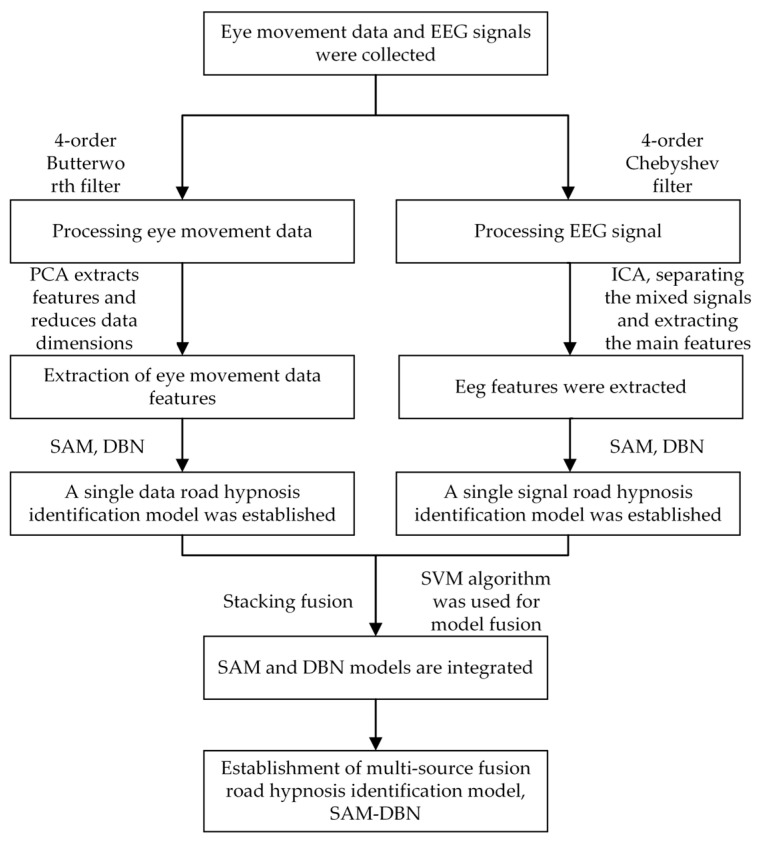
Establishment of road hypnosis identification model.

Models for road hypnosis identification established with EEG physiological signals and eye movement data can both assess the drivers’ road hypnosis state. However, models built with only one type of signal or data feature cannot fully utilize the characteristics of both types of data. In the road hypnosis identification task, EEG signals and eye movement data are two distinct sources of physiological information. After preprocessing and feature extraction, they have different feature representations and data distributions, which makes direct fusion difficult. Therefore, this study chose to use model fusion methods to handle the two types of data with unequal quantities. This approach ensures that the model’s generalization ability is not negatively affected by the limitations of feature fusion techniques. Model fusion can fully utilize the advantages of different models and address the limitations of individual models, which improves overall prediction performance. By combining features from EEG signals and eye movement data, model fusion can capture the drivers’ physiological state and behavioral characteristics more comprehensively, which results in a more robust and generalizable model. Additionally, model fusion can reduce the risk of overfitting and improve the stability and reliability of the road hypnosis identification model. This study used SAM and DBN algorithms to construct models for eye movement data and EEG signals, respectively. The resulting predictions are obtained and then fused using the Stacking method. The specific process is shown in Figure 12.

**Figure 13:**
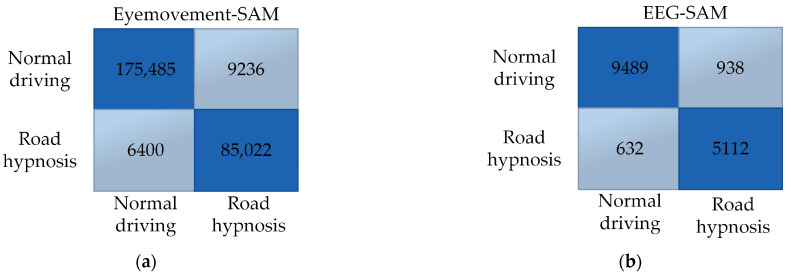

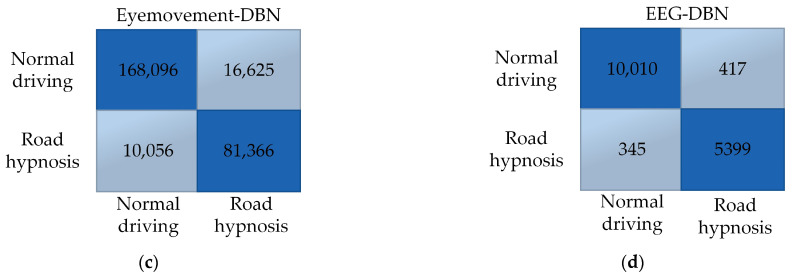
Virtual driving data results. (**a**) Eye movement-SAM. (**b**) EEG-SAM. (**c**) Eye movement-DBN. (**d**) EEG-DBN.

Road hypnosis identification models are established in this study, using eye movement data and EEG signals combined with SAM and DBN algorithms. Models with high accuracy in identifying road hypnosis are fused to improve the effectiveness of road hypnosis detection and better identify drivers’ road hypnosis states. RRSCV is used to validate the models. This method generates multiple training and testing datasets through repeated random subsampling and uses these datasets to estimate the models’ generalization performance. RRSCV ensures that all results are used for training and testing, with each result used once in both processes, allowing for a more accurate assessment of model performance. The resulting confusion matrix is shown in Figure 13:

The superior identification performance of the SAM model constructed with eye movement data compared to the DBN model can be observed through the confusion matrix. The SAM model correctly identifies a greater number of road hypnosis cases compared to the DBN model. For instance, the DBN model incorrectly classified 16,625 normal driving cases as road hypnosis, whereas the SAM model classified 9236 cases incorrectly. This suggests that the SAM model makes fewer errors in identifying road hypnosis as normal driving.

Ten thousand and ten cases of road hypnosis are correctly identified by the DBN model built with EEG data. The SAM model identified 9489 cases of road hypnosis. This indicates that the DBN model with EEG data performs better in identifying road hypnosis compared to the SAM model with the same data. Additionally, the SAM model incorrectly classified 938 normal driving cases as road hypnosis, whereas the DBN model made 417 such errors. This suggests that the DBN model makes fewer errors in misclassifying normal driving as road hypnosis when EEG data are used.

**Figure 14:**
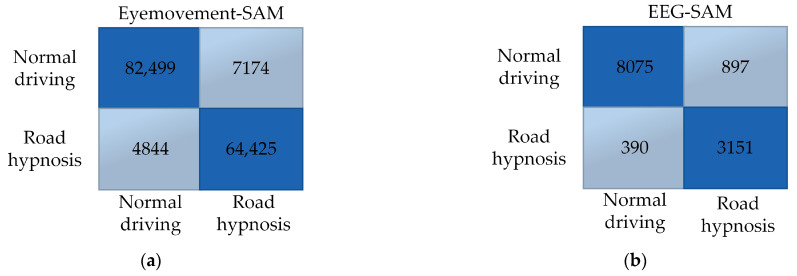

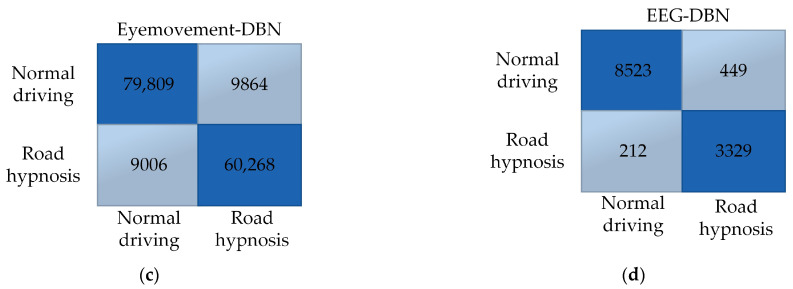
Vehicle driving data results. (**a**) Eye movement-SAM. (**b**) EEG-SAM. (**c**) Eye movement-DBN. (**d**) EEG-DBN.

Overall, the SAM model with eye movement data performs better in virtual driving simulations, while the DBN model with EEG data demonstrates superior performance. The resulting confusion matrix is shown in Figure 14:

In vehicle driving, factors such as environmental complexity, realism, and unstable traffic conditions result in a smaller overall dataset compared to virtual driving experiments. The same judgment methods used in virtual driving show that the SAM model built with eye movement data performs better than the DBN model in vehicle driving. Conversely, the DBN model built with EEG data outperforms the SAM model.

**Figure 15:**
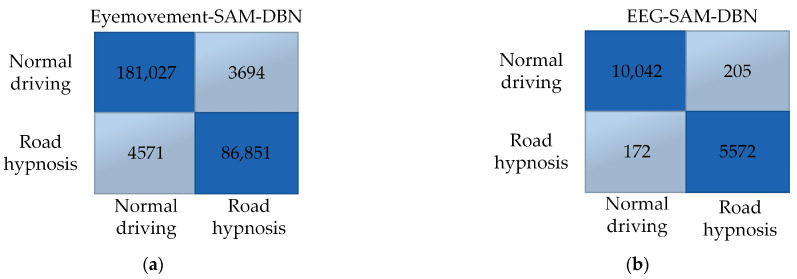
Virtual driving data results. (**a**) Eye movement-SAM-DBN. (**b**) EEG-SAM-DBN.

**Figure 16:**
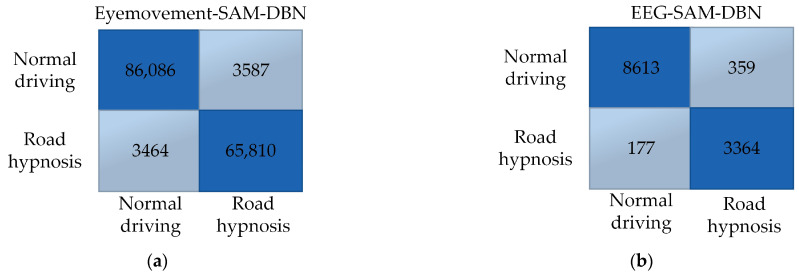
Vehicle driving data results. (**a**) Eye movement-SAM-DBN. (**b**) EEG-SAM-DBN.

In summary, whether in virtual or vehicle driving experiments, the SAM algorithm is more suitable for training with eye movement data, while the DBN algorithm is better suited for EEG data. Therefore, the SAM algorithm should be prioritized for training eye movement data, and the DBN algorithm should be used for training EEG data. The confusion matrix obtained after model fusion is shown in Figure 15 and Figure 16:

Comparisons between the SAM and DBN models reveal that, in both virtual and vehicle driving experiments, the SAM-DBN model identifies more cases of road hypnosis correctly and makes fewer incorrect identifications. This result indicates that the road hypnosis identification model constructed with model fusion performs better.

To further assess the performance and generalization ability of the SAM-DBN model, four metrics are introduced: Accuracy, False Positive Rate (FPR), False Negative Rate (FNR), and Specificity.

Accuracy refers to the ratio of correctly classified samples to the total number of samples. It represents the overall classification accuracy of the classifier. The calculation formula is as follows:

(21) $$\mathrm{Accuracy}=\frac{\mathrm{TP}+\mathrm{TN}}{\mathrm{FP}+\mathrm{TP}+\mathrm{FN}+\mathrm{TN}}\times 100\%$$

In the formulas, TP represents True Positives, TN represents True Negatives, FP represents False Positives, and FN represents False Negatives.

False Positive Rate (FPR) indicates the proportion of actual negative samples incorrectly predicted as positive. A lower FPR is preferable.

(22) $$\mathrm{FPR}=\frac{\mathrm{FP}}{\mathrm{FP}+\mathrm{TN}}\times 100\%$$

False Negative Rate (FNR) denotes the proportion of actual positive samples incorrectly predicted as negative. A lower FNR is preferable.

(23) $$\mathrm{FNR}=\frac{\mathrm{FN}}{\mathrm{FN}+\mathrm{TP}}\times 100\%$$

Specificity measures the proportion of actual negative samples correctly predicted as negative. Specificity assesses the model’s ability to identify negative cases and indicates performance in excluding negatives. A higher Specificity is preferable.

(24) $$\mathrm{Specificity}=\frac{\mathrm{TN}}{\mathrm{TN}+\mathrm{FP}}\times 100\%$$

**Figure 17:**
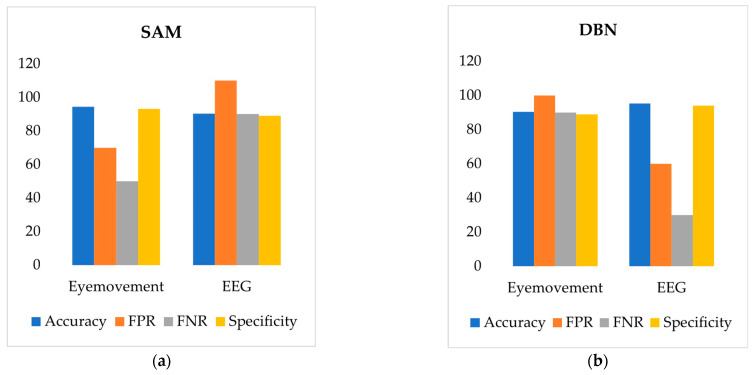
Virtual driving experiment results. (**a**) SAM. (**b**) DBN.

**Figure 18:**
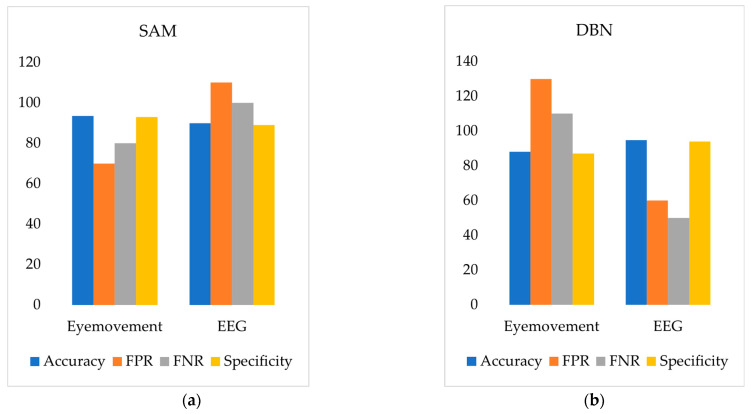
Vehicle driving experiment results. (**a**) SAM. (**b**) DBN.

The experimental results are as follows:

According to Figure 17a and Figure 18a, the SAM model based on eye movement data outperforms the DBN model in both accuracy and specificity while also showing a lower False Positive Rate (FPR) and False Negative Rate (FNR). This indicates that the road hypnosis identification model with the SAM algorithm performs better in identifying road hypnosis. On the other hand, Figure 17b and Figure 18b show that the DBN model based on EEG signals excels over the SAM model in accuracy and specificity, with lower FPR and FNR as well. This suggests that the DBN model provides higher accuracy in identifying road hypnosis during driving.

To further validate the performance and discriminative ability of the SAM-DBN model, it was compared with mainstream deep learning algorithms EEGNet and LSTM. All algorithms were trained and tested on the same dataset to ensure fairness in the comparison.

**Figure 19:**
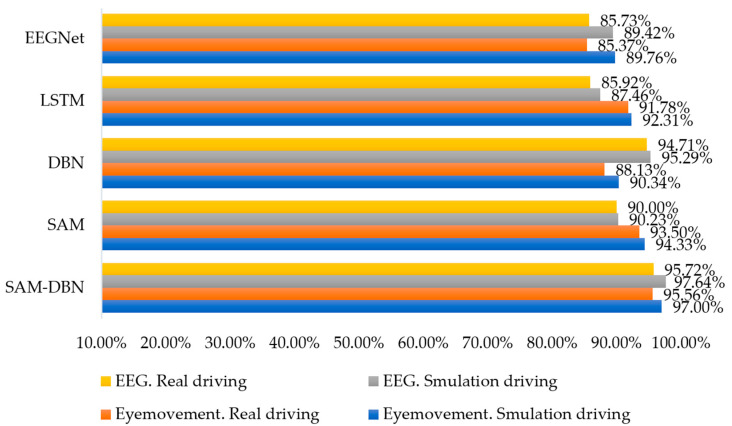
Accuracy of SAM-DBN experiment results.

Figure 19 shows that in both virtual and vehicle driving experiments, the accuracy of the SAM-DBN model is significantly higher when eye movement data and EEG signals are provided as inputs, compared to the SAM, DBN, EEGNet, and LSTM models alone. This result demonstrates that the combination of the SAM and DBN models improves the accuracy of road hypnosis identification more effectively. In addition, the comprehensive application of multi-source data allows for a more complete capture of driver states, which leads to better identification of road hypnosis. However, the accuracy of the model still shows some misidentifications. This is related to the generalization capacity limit of the deep learning model. This limitation is an inherent feature of the deep learning model because the performance of the deep learning model is highly dependent on the distribution of training data. If there are insufficient samples or similarities in features for certain states in the training data, the subtle differences between these states may not be fully captured by the model, which affects its generalization capacity.

Comparing the results from vehicle driving and virtual driving experiments, both datasets can build models with good performance. However, road hypnosis occurs less frequently in vehicle driving experiments, while virtual driving experiments induce road hypnosis more effectively. Although data from vehicle driving are more representative, the overall dataset is smaller and influenced by vehicle driving environments, which leads to slightly lower accuracy compared to virtual driving experiments. A more effective road hypnosis identification model is established in this study through a rigorous and scientifically valid experimental approach combined with EEG signals and eye movement data.

Principal Component Analysis and Independent Component Analysis are used separately in this study to extract features from eye movement and EEG data. Traditional feature fusion techniques, such as concatenation, element-wise addition, and multiplication, are widely used in various deep learning architectures. However, these methods often lack the ability to adapt to specific features of data and models. This limitation leads to suboptimal performance and reduced generalization capability. Therefore, the stacking method is selected in this study to integrate the models trained on different feature data. The predictive abilities of multiple base models are effectively combined, thereby improving the overall performance and generalization ability of the model.

## 4. Conclusions

Vehicle driving experiments and virtual driving experiments are designed in this study. A total of 56 participants were recruited based on driving experience. During the experiments, EEG and eye movement data were collected while drivers were in a state of road hypnosis. After the experiments, data are filtered and removed through video observation and expert scoring. This process lays a foundation for developing an accurate road hypnosis identification model. Butterworth and Chebyshev filters are applied to preprocess eye movement data and EEG signals. Feature extraction occurs with PCA and ICA methods on the preprocessed data. Two algorithms, SAM and DBN, construct road hypnosis identification models. The stacking method integrates models with high prediction accuracy, which results in the multi-source data fusion road hypnosis identification model, SAM-DBN. The effectiveness of the SAM-DBN model is assessed with RRSCV and four metrics: accuracy, false positive rate, false negative rate, and specificity. A comparison of the SAM-DBN model with the SAM, DBN, EEGNet, and LSTM models shows that the SAM-DBN model exhibits superior generalization ability and identification effectiveness.

## Figures and Tables

**Table 1:** Model fusion method.

Model Fusion Method	Definition	Advantages and Disadvantages
Stacking [38]	The final prediction result is obtained by inputting the predictions of multiple models as new features into a meta-model.	This method integrates multiple models in a relatively non-parametric manner and utilizes the advantages of SAM and DBN models to achieve better performance.
Averaging method [39]	Averaging methods include simple averaging and weighted averaging. These methods obtain the final prediction result by averaging or weighted averaging the predictions from multiple models. Weights in weighted averaging can be assigned based on the performance of each model.	Although simple averaging or weighted averaging is an intuitive and easy-to-implement method, it does not account for the differences and complex relationships between models.
Voting method [40]	The final prediction is determined by selecting the category or value with the highest number of votes from multiple models.	The voting method requires consistency among models. However, EEG and eye-tracking data may not be complementary in certain aspects when drivers are in a road hypnosis state, which means they might make different predictions.
Bagging (Bootstrap Aggregating) [41]	Bagging trains multiple models of the same type in parallel, with each model assigned a different training dataset (sampling with replacement). The predictions of these models are then averaged or voted upon.	Both methods allow for the parallel or sequential training of multiple models of the same type, with the final results being combined. However, the models used in this study are of different types and cannot be trained directly in this manner.
Boosting [42]	Boosting is an iterative model fusion method. It involves training a series of weak learners, with each weak learner correcting the errors of the previous one. This process enhances the overall performance of the model.	

## Data Availability

The data presented in this study are available on request from the corresponding author. The data are not publicly available due to privacy.
